# Potential therapeutic benefits of curcumin in depression or anxiety induced by chronic diseases: a systematic review of mechanistic and clinical evidence

**DOI:** 10.3389/fphar.2025.1638645

**Published:** 2025-08-22

**Authors:** Jiyuan Yuan, Chao Pi, Hongping Shen, Bi Zhou, Yumeng Wei, Nathupakorn Dechsupa, Ling Zhao

**Affiliations:** ^1^ Molecular Imaging and Therapy Research Unit, Department of Radiologic Technology, Faculty of Associated Medical Sciences, Chiang Mai University, Chiang Mai, Thailand; ^2^ Luzhou Key Laboratory of Traditional Chinese Medicine for Chronic Diseases Jointly Built by Sichuan and Chongqing, The Affiliated Traditional Chinese Medicine Hospital, Southwest Medical University, Luzhou, Sichuan, China; ^3^ Research Center for Clinical Trials, The Affiliated Traditional Chinese Medicine Hospital, Southwest Medical University, Luzhou, Sichuan, China; ^4^ Central Nervous System Product Research and Development Key Laboratory of Sichuan Province, School of Pharmacy, Southwest Medical University, Luzhou, Sichuan, China

**Keywords:** curcumin, depressive, anxiety, chronic disease, co-occurring disorders

## Abstract

**Introduction:**

Depression and anxiety are prevalent comorbidities in individuals with chronic diseases, significantly impairing their quality of life and complicating disease management. Curcumin, derived from turmeric (Curcuma longa), has garnered attention for its potential therapeutic benefits in alleviating symptoms of depression and anxiety. However, its specific effects on depressive or anxiety symptoms associated with chronic diseases (DACD) remain unclear.

**Methods:**

This review aims to comprehensively evaluate curcumin's efficacy and underlying mechanisms through a meta-analysis of human clinical trials supplemented by findings from animal studies. A systematic literature search was conducted in PubMed, EMBASE, Web of Science, Cochrane Library, EBSCO, and OVID databases (until 25 October 2024), with PROSPERO registration number CRD42024602837. Fifteen randomized controlled trials (RCTs) involving 1,123 adult participants were included.

**Results:**

Meta-analysis showed a statistically significant effect of curcumin on depressive symptoms (SMD: −0.65, P = 0.01, 95% CI: −1.16 to −0.13, I^2^ = 93%) and anxiety symptoms (SMD: −0.22, P = 0.01, 95% CI: −0.40 to −0.05, I^2^ = 0%). Preclinical studies identified several mechanistic pathways through which curcumin may alleviate DACD, including anti-inflammatory and antioxidant effects via NF-κB, NLRP3, AKAP150/PKA/PP2B, miR-146a-5p/ERK, BDNF/TrkB, ROS-ERK1/2, GABA receptors, Keap1-Nrf2-ARE, and regulation of intestinal flora.

**Discussion:**

These findings highlight curcumin's potential in alleviating DACD. However, the conclusions should be interpreted with caution due to considerable heterogeneity across studies, limited sample size, variations in curcumin formulations and dosages, and potential publication bias. Nevertheless, this review provides a comprehensive overview of the current clinical and mechanistic evidence supporting curcumin's role as an adjunctive treatment for depression and anxiety associated with various chronic diseases such as type 2 diabetes, obesity, migraines, arthritis, coronary heart disease, irritable bowel syndrome, inflammatory bowel disease, and metabolic syndromes.

**Systematic Review Registration:**

https://www.crd.york.ac.uk/prospero/display_record.php?ID=CRD42024602837, identifier CRD42024602837.

## 1 Introduction

Numerous studies have explored the psychological wellbeing of patients with chronic diseases, revealing a high prevalence of comorbid depression and anxiety (Scott et al., 2023). Chronic disease like coronary heart disease or diabetes was frequently associated with these mental health disorders, which can exacerbate physical disease progression, increase mortality, and worsen prognosis (Karami et al., 2023; Berk et al., 2023). Estimates suggest that depression affects 9.3%–25% of chronic disease patients (Ma et al., 2021; Uhlenbusch et al., 2021), while anxiety is 1.5–1.8 times more common in this population than in the general public (Uhlenbusch et al., 2019). The coexistence of mental health disorders and chronic physical conditions presents significant clinical and economic challenges, including reduced treatment adherence, greater complexity in disease management, and poorer health outcomes (Glover-Wright et al., 2023; Zeleke et al., 2025). The interplay between mental health disorders and chronic health conditions complicates medical management, resulting in diminished treatment compliance and poorer health outcomes (Abernathy et al., 2016; Alomar et al., 2024).

Several mechanisms have been proposed to underlie depression and anxiety, including alterations in monoaminergic and glutamatergic neurotransmission, deficits in neuroplasticity, hypothalamic-pituitary-adrenal (HPA) axis dysregulation, and elevated levels of neuroinflammation and oxidative stress (Chen et al., 2023; Wiebenga et al., 2022). Current first-line pharmacological treatments for depression are primarily based on the monoaminergic hypothesis. These include selective serotonin reuptake inhibitors (SSRIs), serotonin and norepinephrine reuptake inhibitors (SNRIs), norepinephrine and specific 5-hydroxytryptamine antidepressants, as well as benzodiazepines (Brandt et al., 2024; Zelek-Molik and Litwa, 2025). However, these medications have several important limitations: (1) delayed onset of therapeutic action, typically requiring weeks to months for clinical effect; (2) limited efficacy, with only 30%–40% of patients achieving full remission; (3) a broad range of side effects, such as drowsiness, dizziness, dry mouth, constipation, weight gain, and sexual dysfunction; and (4) risk of discontinuation syndromes, where abrupt withdrawal may lead to headaches, nausea, and rebound anxiety (Sorensen et al., 2022; Lagerberg et al., 2023; Fornaro et al., 2019; Protti et al., 2020).

Fortunately, recent studies have shown that many active compounds found in natural drugs have promising therapeutic effects on depression and anxiety and are considered highly safe (Wu et al., 2022; Yang et al., 2024). One notable example is curcumin, which is a polyphenolic compound derived from turmeric, a spice known for its culinary uses. Curcumin has garnered extensive research attention for its medicinal properties (Lopresti, 2022). In addition to supporting standard therapy and promoting a healthy lifestyle, curcumin has been found to improve a variety of health conditions, including cancer, osteoarthritis, migraines, diabetes, nervous system disorders, metabolic syndrome, oral diseases, and others, thanks to its antioxidant and anti-inflammatory properties (Hassanizadeh et al., 2023; Kumar et al., 2021; Patel et al., 2020).

A critical question remains: Can curcumin alleviate depression or anxiety symptoms arising from chronic diseases (DACD)? Despite scholars conducting relevant research, no comprehensive review has yet consolidated the available evidence to draw definitive conclusions. To address this gap, the present study adopts a dual approach: (1) evaluating preclinical (animal) studies to elucidate the mechanisms by which curcumin may relieve DACD-related emotional symptoms, and (2) conducting a meta-analysis of human clinical trials to assess its therapeutic efficacy. By integrating both mechanistic and clinical evidence, this review aims to improve understanding of curcumin’s potential role and inform its future application in the management of DACD.

## 2 Potential mechanism of curcumin in DACD

### 2.1 Obesity and type 2 diabetes mellitus (T2DM)

Obesity and type 2 diabetes mellitus (T2DM) are frequently accompanied by depression and anxiety, which together exacerbate disease progression and complicate management (American Diabetes, 2021). Epidemiological evidence indicates that individuals with obesity or T2DM are significantly more likely to experience depression or anxiety compared to the general population (Dakanalis et al., 2023; Li et al., 2024). The bidirectional relationship between metabolic dysfunction and depression is increasingly supported by mechanistic evidence. Hyperglycemia and dyslipidemia promote depressive symptoms by increasing systemic inflammation and reducing brain levels of serotonin (5-hydroxytryptamine, 5-HT) (Cai et al., 2022; Chourpiliadis et al., 2024). In T2DM, insulin signaling dysregulation further disrupts 5-HT neurotransmission, creating a bidirectional relationship between metabolic dysfunction and depression (Khawagi et al., 2024). Notably, depression itself exacerbates hyperglycemia and complicates glycemic control, establishing a vicious cycle. Curcumin reduces the production of pro-inflammatory cytokines, such as TNF-α and IL-6, which are central to the pathophysiology of diabetes and obesity (Hussain et al., 2022). By attenuating inflammation and oxidative stress, curcumin improves insulin receptor signaling and enhances insulin sensitivity—mechanisms that are particularly relevant given the shared inflammatory and insulin-resistant states underlying diabetes and depression (Sarmiento-Ortega et al., 2025). Curcumin modulates critical pathways such as NF-κB and downregulates IL-6 and TNF-α signaling, leading to improvements in both glucose metabolism and depressive symptoms (Sarmiento-Ortega et al., 2025; Su et al., 2017; Fan et al., 2018; Lee and Kim, 2024).

Meanwhile, insulin resistance (IR) not only co-occurs with depressive symptoms but also contributes to poor antidepressant treatment response and is closely associated with metabolic subtype depression—representing a shared biological mechanism underlying both depression and T2DM (Khawagi et al., 2024; Krupa et al., 2024). In a rat model of IR-related depression induced by chronic mild stress (CMS), Shen et al. (Shen et al., 2017) demonstrated that curcumin upregulated hepatic insulin receptor substrate (IRS)-1 and Akt phosphorylation, thereby enhancing insulin sensitivity. Notably, curcumin simultaneously alleviated depressive-like behaviors and reversed CMS-induced metabolic disturbances. These findings provide compelling evidence for curcumin’s dual therapeutic potential in modulating both mood symptoms and metabolic dysfunction associated with insulin resistance.

Furthermore, in a large meta-analysis (Wang et al., 2021), long-term use of antidepressants was associated with an increased risk of new-onset type 2 diabetes, particularly for Tricyclic antidepressants (TCAs). In pancreatic β-cells (RINm5F), curcumin prevents desipramine (which belongs to TCAs) induced apoptosis by inhibiting the PI3K/AKT/FOXO1 pathway and disrupting AKAP150/PKA/PP2B interactions. These findings position curcumin as a potential adjunct therapy that could preserve β-cell function and improve insulin regulation during antidepressant treatment (Hu et al., 2024). This was emerging evidence that suggests curcumin may counteract some adverse metabolic effects of antidepressants.

### 2.2 Cardiovascular and cerebrovascular diseases

Post-stroke depression (PSD) is a prevalent and clinically significant neuropsychiatric complication following stroke (Liu et al., 2023). Its pathophysiology involves multiple interconnected mechanisms, including monoaminergic neurotransmitter depletion, impaired neurotrophic signaling, neuroinflammation accompanied by hypothalamic-pituitary-adrenal (HPA) axis dysregulation, and glutamate-induced excitotoxicity (Medeir et al., 2020). Curcumin has shown promising neuroprotective effects in this context. In lipopolysaccharide (LPS)-induced depressive rat models, curcumin pre-treatment reduced neuronal death in the hippocampal CA1 region, likely via the miR-146a-5p/ERK signaling pathway, and was associated with improved synaptic function (Fan et al., 2021). Additionally, curcumin exhibits dose-dependent neuromodulatory activity—enhancing 5-HT levels and, at higher concentrations, increasing dopamine availability through dual inhibition of monoamine oxidase A and B (MAO-A and MAO-B) (Fang et al., 2022). Recent studies also suggest that curcumin upregulates long noncoding RNA growth arrest-specific transcript 5 (GAS5), which activates the brain-derived neurotrophic factor (BDNF)/TrkB signaling pathway to promote synaptic protein expression. By suppressing miR-10b, GAS5 upregulation enhances BDNF mRNA levels, contributing to curcumin’s antidepressant effects in PSD via the GAS5/miR-10b/BDNF regulatory axis (Cai et al., 2020).

Patients with myocardial infarction (MI) exhibit significantly increased risks of developing depression and anxiety disorders (Sun et al., 2021; Yang et al., 2021; Ryan et al., 2022). Post-MI depression notably reduces quality of life and is associated with a 2- to 2.5-fold increase in cardiovascular mortality (Garrels et al., 2023). Physiological stress after MI activates the HPA axis, increasing CRH, ACTH, and glucocorticoid levels. Chronic HPA hyperactivity is considered a key mechanism in post-MI depression (Yang et al., 2021). Curcumin exerts antidepressant effects, likely via modulation of the HPA axis and neuroprotective pathways, while also reducing myocardial injury by suppressing cellular necrosis and apoptosis (Yang et al., 2021). Additionally, curcumin significantly inhibits NLRP3 inflammasome activation through its antioxidant properties, thereby improving mitochondrial function and reducing reactive oxygen species (ROS) production. This, in turn, alleviates neuroinflammation and depressive-like behaviors in chronic stress models, such as chronic unpredictable mild stress (Du et al., 2023). Further supporting a neuroendocrine link, Monticone et al. observed that treatment of primary aldosteronism—via adrenalectomy or mineralocorticoid receptor antagonists—significantly reduced depressive symptoms only when aldosterone levels were normalized, indicating a mechanistic relationship between aldosterone excess and depression (Murck et al., 2021). In line with this, Zhang et al. demonstrated that curcumin suppresses aldosterone-induced C-reactive protein (CRP) production in vascular smooth muscle cells by inhibiting the ROS-ERK1/2 signaling pathway (Zhang et al., 2020a).

### 2.3 Chronic pain

National Health Interview Survey (U.S.) 2019 data showed that individuals with chronic pain had significantly elevated odds of experiencing moderate-to-severe depressive or anxiety symptoms (Mullins et al., 2023). This bidirectional relationship is clinically significant as anxiety and depression exacerbate pain perception, leading to poorer treatment outcomes, more severe symptoms, and reduced quality of life (Roughan et al., 2021). Both clinical and preclinical studies consistently identify oxidative stress as a key mediator in the pain-depression cycle (Yang et al., 2020). Arora et al. demonstrated that curcumin dose-dependently alleviated pain-depression behaviors. This therapeutic effect was mechanistically linked to reductions in pro-inflammatory cytokines (TNF-α, IL-1β), enhancement of antioxidant defenses (e.g., increased superoxide dismutase activity), suppression of apoptosis via caspase-3 inhibition, and modulation of NF-κB pathways—collectively yielding neuroprotection and symptomatic relief (Arora et al., 2011). These findings provide strong experimental evidence that curcumin targets oxidative stress and inflammatory cascades to mitigate the intertwined pathophysiology of pain and depression.

Curcumin shows therapeutic potential for managing peripheral neuropathic pain through multiple mechanisms (Basu et al., 2021). Its anti-inflammatory effects in microglia and astrocytes stem from inhibiting key pro-inflammatory mediators by blocking MAPK, NF-κB, and JAK-STAT pathways, thereby reducing pain hypersensitivity and spontaneous pain (Hasriadi et al., 2021). Experimental studies demonstrate curcumin alleviates oxaliplatin-induced neuropathic pain by suppressing oxidative stress-mediated NF-κB activation and subsequent inflammation (Zhang et al., 2020b). In trigeminal neuralgia models, curcumin ameliorates chronic pain-induced depression by modulating lipid and glycerophospholipid metabolism-potentially key pathways in pain-depression comorbidity (Zhang L. et al., 2020). The compound exerts dual benefits in neuropathic mice, normalizing depression-like behaviors through supraspinal serotonergic modulation (enhancing 5-HT signaling) and GABAA receptor activation while simultaneously reducing neuropathic pain symptoms (Zhao et al., 2014). Although clinical evidence supports curcumin’s efficacy against depression/anxiety in patients with bone/joint pain, corresponding animal studies remain lacking.

### 2.4 Neurological diseases

Neurological diseases (NDs) have become the leading global cause of disability-adjusted life years, with a substantially increased burden since 1990 (Ding et al., 2022). These disorders frequently present with depression and anxiety symptoms due to shared neural, inflammatory, and biochemical mechanisms (Ray and Agarwal, 2020; Botto et al., 2022; Gregory et al., 2017; Johnson et al., 2024). Experimental evidence demonstrates curcumin’s neuroprotective effects through multiple pathways. Khan et al. showed dietary curcumin prevents LPS-induced neuroinflammation and cognitive impairment in rats by modulating JNK/NF-κB/Akt signaling (Khan et al., 2019). Curcumin supplementation has been shown to restore brain-derived neurotrophic factor (BDNF) levels while regulating monoaminergic neurotransmission and mitigating multiple pathological processes including oxidative stress, neuroinflammation, β-amyloid aggregation, tau pathology, and aluminum-induced neurotoxicity in both Alzheimer’s disease and depression models (Alam et al., 2024). Notably, curcumin’s potent antioxidant properties modulate stress responses, attenuating anxiety-like behaviors while preserving the cognitive-enhancing effects of acute stress (Haider et al., 2015). This dual modulation suggests curcumin may optimize physiological stress responses when administered as a nutritional supplement.

Current research underscores curcumin’s neuroprotective properties and its potential synergy with cognitive-behavioral therapy for managing depression and anxiety in Parkinson’s disease (PD) patients (Singh et al., 2020; Bh et al., 2019; Josifovska et al., 2023). Emerging evidence suggests that novel curcumin-based compounds, either alone or in combination with anti-inflammatory drugs, may represent optimal therapeutic strategies for both major depression and PD (Tizabi et al., 2014). Chronic manganese exposure is known to cause progressive neurological damage with PD-like symptoms (Pajarillo et al., 2022). Recent studies demonstrate that targeted curcumin administration mitigates striatal dopaminergic neuron damage in manganese-exposed rats, improving neurobehavioral outcomes while reducing α-Synuclein aggregation and apoptosis. These neuroprotective effects appear mediated through enhanced cellular autophagy via TFEB nuclear translocation (Lai et al., 2022). Furthermore, in rotenone-induced PD models (an established environmental toxin linked to increased PD risk), curcumin has shown significant antidepressant activity against toxin-induced depressive behaviors (Madiha and Haider). These findings collectively highlight curcumin’s multifaceted therapeutic potential across various PD-related pathologies.

Current evidence indicates that curcumin exerts protective effects against epileptic seizures and associated memory impairment, potentially through modulation of brain monoamine levels (Forouzanfar et al., 2021). Research demonstrates that when formulated as solid lipid nanoparticles, curcumin shows enhanced neuroprotective activity against epilepsy by activating both Bcl-2 family proteins and p38 MAPK signaling pathways (Huang et al., 2020). Further supporting these findings, Choudhary et al. established that curcumin administration reduces seizure severity while improving depression-like behaviors and cognitive deficits in murine models. These therapeutic benefits appear to result from curcumin’s ability to balance excitatory and inhibitory neurotransmitter systems, thereby decreasing neuronal hyperexcitability associated with epileptic conditions (Khatoon and Kalam, 2024).

### 2.5 Digestive disorders

Irritable bowel syndrome (IBS) affects 5%–10% of the global population (Staudacher et al., 2023), and frequently co-occurs with stress-related psychiatric disorders such as depression and anxiety (Staudacher et al., 2023). IBS patients typically exhibit elevated brain-derived neurotrophic factor (BDNF) levels (Konturek et al., 2020). Preclinical studies demonstrate curcumin’s dual therapeutic effects in IBS models. Research by Yu et al. demonstrated that curcumin administration produced multiple beneficial effects in IBS rat models, including normalization of visceral hypersensitivity responses to colorectal distension, improvement in stool consistency, and reduction of depressive-like and anxiety-like behaviors. These therapeutic effects appear to be mediated through curcumin’s modulation of key neuromodulators, with particularly noteworthy differential regulation observed between brain and gut tissues: while BDNF and phosphorylated CREB (p-CREB) levels increased in hippocampal tissue, their expression decreased in colonic tissues (Yu et al., 2015).

Clinical evidence indicates that anxiety and depression represent the most prevalent psychiatric comorbidities in inflammatory bowel disease (IBD) patients, significantly impairing both quality of life and disease progression (Yamamoto-Furusho et al., 2021). Emerging research highlights the crucial role of gut microbiota in this relationship, particularly the *Muribaculaceae* family. These commensal bacteria colonize the intestinal mucus layer, metabolizing aminoglycans to produce short-chain fatty acids (SCFAs) while stimulating mucin secretion and activating anti-inflammatory pathways (Zhu et al., 2024; van der Hee and Wells, 2021). Recent mechanistic studies by Zhang et al. demonstrate that curcumin modulates this gut-brain axis by increasing prefrontal cortex phosphatidylcholine levels and alleviating Dextran sulfate sodium (DSS)-induced anxiety behaviors through targeted microbiota alterations. Their work specifically identifies *Muribaculaceae* as a key microbial mediator in curcumin’s anxiolytic effects via the microbiota-gut-brain (MGB) axis (Zhang et al., 2022).

### 2.6 Others

Current research demonstrates curcumin’s significant neuroprotective properties against various chemotherapy agents. Liao et al. showed that curcumin effectively counteracts doxorubicin (DOX)-induced neurotoxicity by suppressing autophagy while reducing oxidative and endoplasmic reticulum stress, with these protective effects mediated through activation of the Keap1-Nrf2-ARE signaling pathway. This mechanism concurrently alleviates DOX-associated depressive-like behaviors (Liao et al., 2023). Similarly, curcumin shows promise as an adjuvant therapy for cisplatin treatment. As a widely used platinum-based chemotherapeutic, cisplatin frequently causes dose-limiting emotional disturbances. Experimental evidence indicates curcumin safely mitigates these effects, reducing cisplatin-induced anxiety and depressive behaviors (Demir et al., 2016). Furthermore, studies reveal curcumin’s perinatal protective effects against mercury toxicity. In developing mouse offspring exposed to mercuric chloride (HgCl2), curcumin administration significantly attenuated anxiety and depression-like behaviors while normalizing biochemical markers of toxicity (Mohammad Abu-Taweel and Al-Fifi, 2021).

Emerging meta-analyses suggest a potential association between *T. gondii* infection (as indicated by IgG seropositivity) and increased risk of anxiety disorders and depression, though current evidence remains correlational. Experimental studies demonstrate curcumin’s neuroprotective effects in *Toxoplasma gondii*-infected neural precursor cells (NPCs) derived from embryonic mouse telencephalon following maternal infection with the VEG strain (de Bles et al., 2021).

**FIGURE 1:**
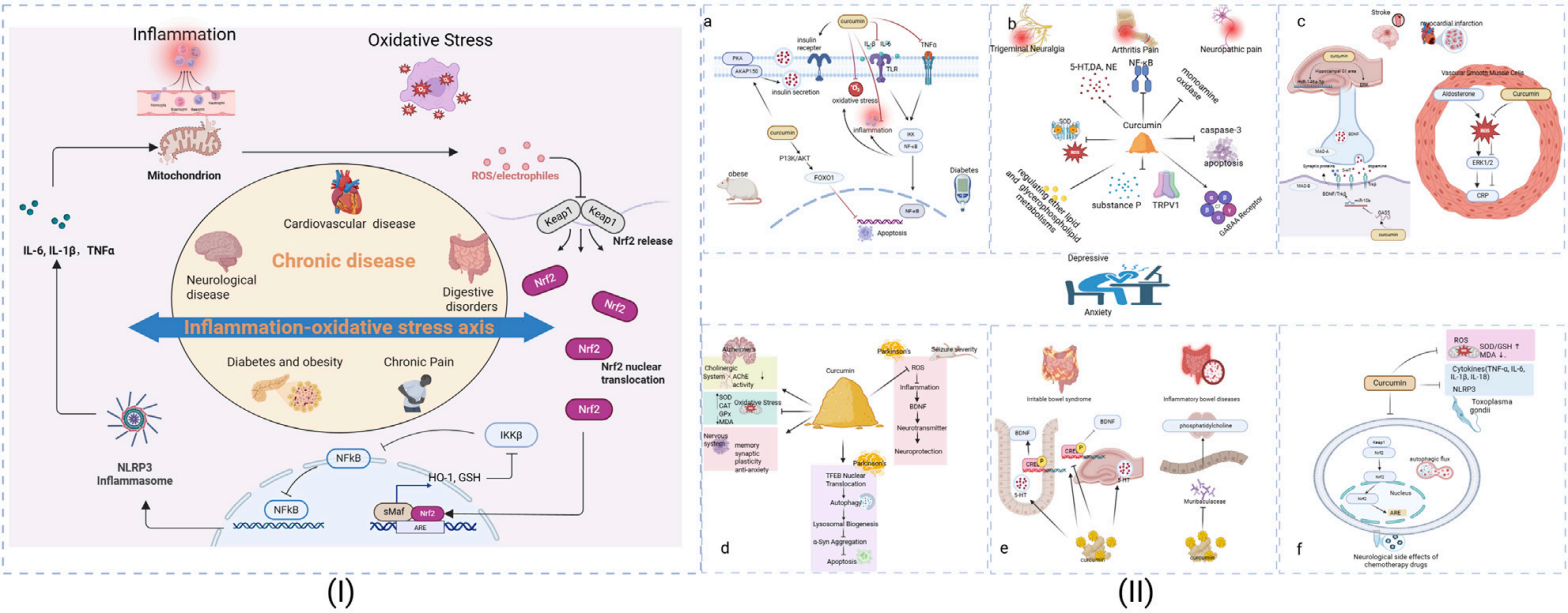
The Analysis of the possible mechanism of curcumin on depression/anxiety in patients with chronic diseases. (I): The inflammation-oxidative stress axis across chronic conditions. (II): **(a)** For Obese and Diabetes, **(b)** For Trigeminal Neuralgia, Arthritis and Neuropathic pain, **(c)** For Stroke and Myocardial infarction, **(d)** For Alzheimer, Parkinson and Seizure severity, **(e)** For Irritable bowel syndrome and Inflammatory bowel disease, **(f)** Neurological side effects of Chemotherapy drugs. Superoxide Dismutase (SOD), Catalase (CAT), Glutathione Peroxidase (GPx), Glutathione (GSH), Heme oxygenase-1(HO-1).

Curcumin treatment effectively restored NPC morphology while modulating key inflammatory pathways. Infected cells showed elevated ERK1/2 expression, which was normalized following curcumin administration, suggesting anti-inflammatory activity (Bissacotti et al., 2023). Mechanistically, curcumin appears to target *T. gondii* detoxification pathways while also addressing infection-associated oxidative stress. Infected mice exhibit characteristic anxiety and depressive-like behaviors correlated with reduced antioxidant enzymes (SOD, GSH) and elevated malondialdehyde (MDA) levels, indicating ROS-mediated lipid peroxidation. Notably, curcumin ameliorates these behavioral symptoms by restoring redox balance and reducing hippocampal proinflammatory cytokines (Moradi et al., 2023). The proposed mechanisms underlying the antidepressant and anti-colitis effects of the extract are summarized in Figure 1.

## 3 Meta-analysis of random trials

### 3.1 Materials and methods

#### 3.1.1 Search strategy

This systematic review was conducted in accordance with PRISMA guidelines and prospectively registered in PROSPERO (*CRD42024602837*) on 17 October 2024. A comprehensive literature search was performed across multiple databases, including Web of Science, PubMed, Ovid, EMBASE, Cochrane Library, and EBSCO without time restrictions, encompassing all randomized controlled trials published in English or Chinese from database inception through 25 October 2024. The search terms used were: Curcumin, Yellow Turmeric, Curcumin Phytosome, Curcuma, Phytosome Curcumin, Diferuloylmethane, turmeric, curcuminoids, Depression, Depressive symptoms, Depressive Symptom, Symptom Depressive, Emotional Depression, Depression, Emotional, major depressive disorder, Angst; Nervousness; Hypervigilance; Social Anxiety; Anxieties, Social; Anxiety, Social; Social Anxieties; Anxiousness. To ensure comprehensive coverage, reference lists of relevant meta-analyses were manually screened for additional eligible studies. All identified records were imported into EndNote X9 (Clarivate™, Version 9.3.3) for duplicate removal and systematic management prior to the screening process.

#### 3.1.2 Eligibility criteria

This meta-analysis exclusively incorporated randomized controlled trials (RCTs) meeting the following inclusion criteria: (1) published clinical trials employing randomization (not restricted to double-blind designs); (2) interventions comparing curcumin at any dosage against either placebo plus standard care or standard care alone; (3) study populations comprising patients with chronic diseases exhibiting depressive or anxiety symptoms, as measured by validated assessment scales; and (4) reported outcomes including both treatment efficacy and safety parameters. We systematically excluded observational studies, literature reviews, *in vitro*/*ex vivo* investigations, quasi-experimental designs, conference proceedings, book chapters, pediatric studies, and publications lacking essential methodological details. The screening process was conducted using EndNote software (Clarivate), with duplicate records automatically identified and removed prior to full-text evaluation.

#### 3.1.3 Data extraction

Two independent investigators (YYJ and PC) conducted the data extraction process using a pre-established extraction protocol to evaluate study quality and collect required data. When reported data were incomplete, we employed either graphical data extraction techniques or contacted the corresponding authors for clarification. All discrepancies between reviewers were resolved through consensus-based discussions with a senior researcher (LZ).

#### 3.1.4 Measures of treatment effect

For studies with missing data, we contacted corresponding authors via email to request necessary information. When author responses were not obtained, we calculated missing summary statistics (including standard deviations of depressive symptom scores) using established methodologies from the Cochrane Handbook for Systematic Reviews of Interventions (Yaikwawong et al., 2024) (Soltani et al., 2024).

#### 3.1.5 Statistical analysis

The primary efficacy outcome was determined by the standardized mean difference (SMD) in validated symptom scale scores between baseline and post-treatment assessments. Secondary outcomes included treatment discontinuation rates (defined as premature withdrawal from the trial for any reason before study completion in either the curcumin or placebo groups) and incidence of adverse events (characterized as any unfavorable medical occurrences attributable to trial participation). All data was conducted using STATA (version 15.1) and Review Manager software. Continuous outcome data, expressed as mean ± standard deviation (SD), were analyzed to calculate pooled effect sizes presented as standardized mean difference (SMD) with 95% confidence intervals (CI). For dichotomous outcomes (dropout rates and adverse events), we computed pooled odds ratios (OR). Given anticipated clinical and methodological heterogeneity across studies, we employed random-effects models for all analyses. Study heterogeneity was evaluated using I^2^ statistics, with values exceeding 50% or Cochran’s Q-test p-values <0.1 indicating substantial heterogeneity, consistent with Cochrane guidelines (Cumpston et al., 2019).

We evaluated potential publication bias using funnel plot asymmetry analysis and Egger’s regression test (Egger et al., 1997) when the analysis included ten or more randomized controlled trials. We conducted stratified subgroup analyses based on several clinically relevant factors: study methodological quality (risk of bias assessment), participant characteristics including depressive symptoms with chronic disease (DCD), anxiety symptoms with chronic disease (ACD), body mass index (BMI) categories, intervention duration variations, treatment protocol differences, and baseline health status variations. To test the robustness of the observations, the following sensitivity analysis was performed: (1) employed a leave-one-out approach, (2) calculated a fixed-effects model, and (3) excluded the trials with a high risk of bias.

### 3.2 Results

#### 3.2.1 Selected studies

**FIGURE 2:**
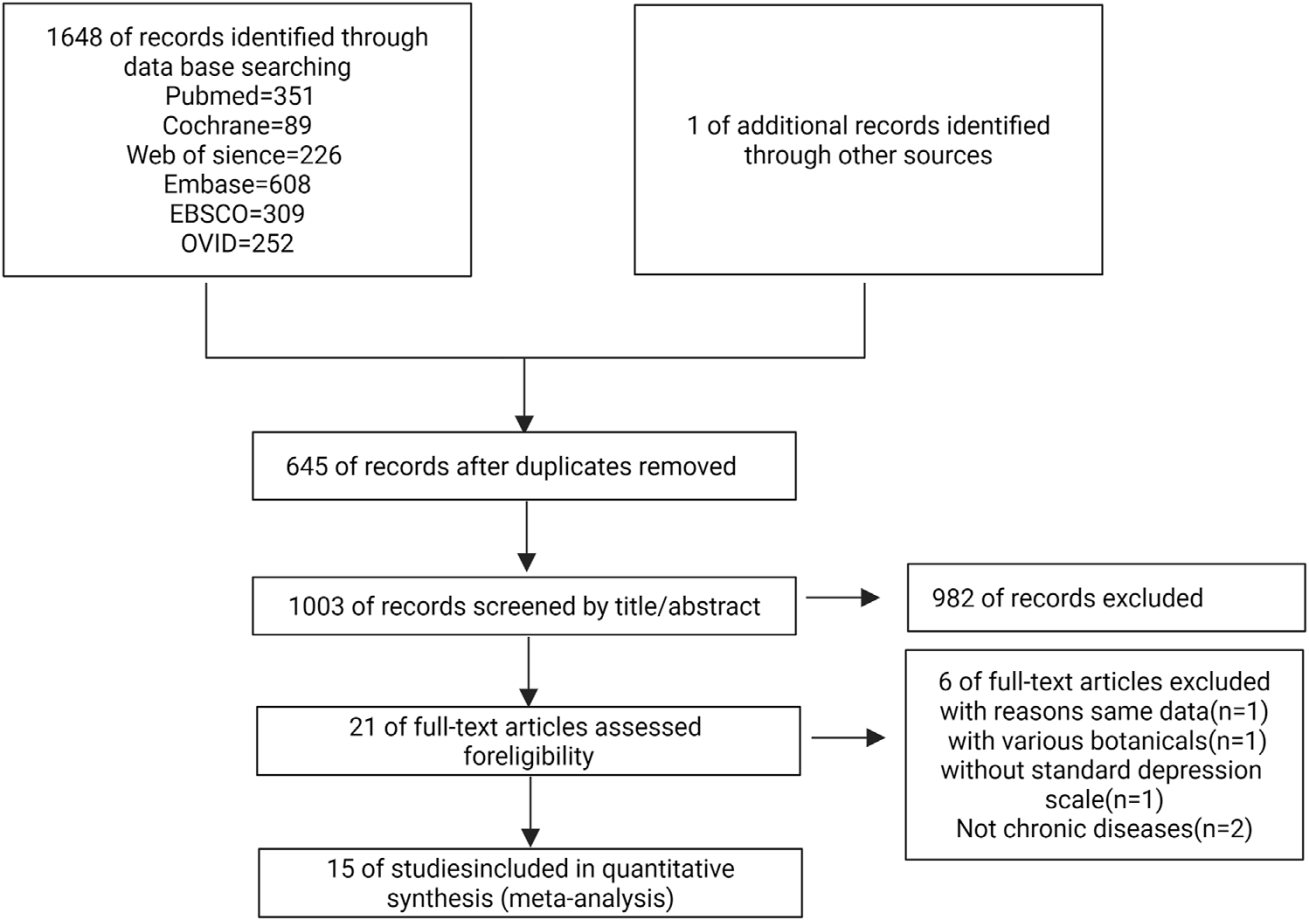
Flow diagram of study selection.

Our systematic search identified 1,648 potentially relevant studies, with one additional article obtained through author correspondence. Following independent review by two investigators, 15 randomized controlled trials (RCTs) involving 1,123 participants met eligibility criteria and were included in the final systematic review and meta-analysis. The complete study selection process, including reasons for exclusion at each screening stage, is detailed in the PRISMA flowchart (Figure 2).

#### 3.2.2 Main characteristics

The fifteen RCTs (Yaikwawong et al., 2024; Soltani et al., 2024; Asadi et al., 2020; Esmaily et al., 2015; Hosseininasab et al., 2021; Kuszewski et al., 2020a; Lopresti et al., 2021a; Lopresti et al., 2021b; Miodownik et al., 2019; Mojtahedi et al., 2024; Osali and Rostami, 2023; Saberi-Karimian et al., 2018; Setiawati et al., 2017; Shafabakhsh et al., 2020; Hosseini et al., 2024) counted depressive symptoms with chronic disease (DCD), which contained an aggregate of 1,123 adult patients, composed of 551 curcumin and 539 placebo patients. Seven articles (Asadi et al., 2020; Lopresti et al., 2021a; Lopresti et al., 2021b; Mojtahedi et al., 2024; Saberi-Karimian et al., 2018; Shafabakhsh et al., 2020; Hosseini et al., 2024) counted anxiety symptoms with chronic disease (ASD), which contained an aggregate of 494 adult patients, composed of 249 curcumin and 245 placebo patients. Two studies (Esmaily et al., 2015; Kuszewski et al., 2020a) had a crossover design, while the others had a parallel-group design. BMI of patients in ≥24.8, including different chronic diseases with depression or anxiet, and the prime diagnoses were obese (Yaikwawong et al., 2024; Esmaily et al., 2015; Lopresti et al., 2021a), diabetes (Yaikwawong et al., 2024; Asadi et al., 2020; Shafabakhsh et al., 2020; Hosseini et al., 2024), metabolic syndrome (Osali and Rostami, 2023; Saberi-Karimian et al., 2018), gastrointestinal symptoms (Lopresti et al., 2021b), Osteoarthritis (Lopresti et al., 2021a), migraine (Mojtahedi et al., 2024) and coronary slow flow phenomenon (Soltani et al., 2024), respectively. The intervention drug used in the three studies (Esmaily et al., 2015; Lopresti et al., 2021a; Lopresti et al., 2021b) was C3 Complex^®^ (consists of curcumin, demethoxycurcumin and bisdemethoxy-curcumin); in five studies (Soltani et al., 2024; Asadi et al., 2020; Hosseininasab et al., 2021; Mojtahedi et al., 2024; Osali and Rostami, 2023) were nanocurcumin capsule; in one study (Saberi-Karimian et al., 2018) was phospholipidated curcumin compared to curcmin and placebo; in one study (Setiawati et al., 2017) was the curcumin which extract obtained from Curcuma xanthorrhiza Roxb; in one study (Yaikwawong et al., 2024) was curcumin capsule contained 250 mg of curcuminoids. Piperine (Esmaily et al., 2015; Miodownik et al., 2019; Hosseini et al., 2024) was used to increase curcumin bioavailability in three studies. The dosages ranged from 80 mg daily to 3000 mg daily. None of the studies used other antidepressants or anxiety medications. Control groups were treated with placebo capsules except in one study (Osali and Rostami, 2023), which set exercise as the control. Different studies have used different questionnaires: Depression, Anxiety, Stress Scale (DASS-21-items) (Asadi et al., 2020; Lopresti et al., 2021b; Mojtahedi et al., 2024; Hosseini et al., 2024), Beck Anxiety Inventory (BAI) (Esmaily et al., 2015; Saberi-Karimian et al., 2018), Beck Depression Inventory (BDI) (Esmaily et al., 2015; Osali and Rostami, 2023; Saberi-Karimian et al., 2018; Setiawati et al., 2017; Shafabakhsh et al., 2020), Beck’s depression inventory-II (BDI-II) (Soltani et al., 2024), Depression Scale for Schizophrenia (CDSS) (Hosseininasab et al., 2021; Miodownik et al., 2019; Saberi-Karimian et al., 2018), Centre for Epidemiologic Studies Depression Scale (CES-D) (Kuszewski et al., 2020a), Patient-Reported Outcomes Measurement Information System-29 (PROMIS-29) (Lopresti et al., 2021a), Patient Health Questionnaire (PHQ-9) (Yaikwawong et al., 2024), The characteristics of the included studies are shown in Table 1.

**TABLE 1:** Characteristics of the included studies.

No	Design	Pts, n	Diagnose	Age, years/gender	Baseline BMI	Locat-ion	Duration	Tx	Bioenh-ancers	Ctrl	Scale
1 (Asadi et al., 2020)	DB, parallel	Int:40 Ctrl:40	diabetic patients with peripheral neuropathy	Int:53.3 ± 6.5 Ctrl:54.6 ± 6.2 Both	Int:31.1 ± 4.2 Ctrl:30.8 ± 3.8	Iran	8 weeks	80 mg/day Nano‐curcumin		PBO	DASS-21
2 (Esmaily et al., 2015)	DB, cross-over	Int:15 Ctrl:15	Obese	Int:38.84 ± 11.12 Ctrl:37.81 ± 12.31 Both	Int:33.95 Ctrl:32.66	Iran	30 days	1 g/day Curcuminoids	Piperine	PBO	BAI BDI
3 (Hosseini et al., 2024)	DB, parallel	Int:33 Ctrl:32	T2DM and hypertriglyceridemia	Int:55.25 ± 7.46 Ctrl:56.17 ± 6.17 Both	Int:31.55 ± 6.42 Ctrl:30.55 ± 6.02	Iran	12 weeks	500 mg/day Curcumin		PBO	DASS-21
4 (Hosseininasab et al., 2021)	DB, parallel	Int:30 Ctrl:32	Chronic Schizophrenia	Int:49.3 ± 11.7 Ctrl:47.8 ± 12.1 Both	NR	Iran	16 weeks	160 mg/day Nanocurcumin		PBO	CDSS
5 (Kuszewski et al., 2020a)	DB 2 × 2 factorial	Int:30 Ctrl:32	Older adults with overweight /obesity	Int:65.4 ± 1.2 Ctrl:65.4 ± 1.3 Both	Int:30.5 ± 0.7 Ctrl:31.0 ± 0.7	AU-NZ	16 weeks	160 mg/day curcumin		PBO	CES-D
6 (Lopresti et al., 2021a)	DB, parallel	Int:51 Ctrl:47	Osteoarthritis Pain of the Knee	Int:59.59 ± 6.57 Ctrl:57.92 ± 6.03 Both	Int:28.82 ± 4.6 Ctrl:28.9 ± 4.1	AU-NZ	8 weeks	1,000 mg/day Curcuminoids	piperine	PBO	PROMIS-29
7 (Lopresti et al., 2021b)	DB, parallel	Int:35 Ctrl:35	Gastrointestinal symptoms and intestinal microbiota	Int:40.69 ± 10.9 Ctrl:43.20 ± 10.3 Both	Int:25.5 ± 3.9 Ctrl:24.8 ± 3.8	AU-NZ	8-week	500 mg/day Curcuminoids		PBO	DASS-21
8 (Miodownik et al., 2019)	DB, parallel	Int:20 Ctrl:18	Chronic Schizophrenia	Int:54.1 ± 12.9 Ctrl:53.4 ± 14.9 Both	NR	Israel	24-week	3,000 mg/day curcumin	piperine	PBO	CDSS
9 (Mojtahedi et al., 2024)	DB, Parallel	Int:22 Ctrl:20	Migraine	Int:39.27 ± 2.15 Ctrl:41.00 ± 2.42 Both	Int:29.66 ± 0.86 Ctrl:29.03 ± 0.75	Iran	2 months	80 mg/day nano-curcumin		PBO	DASS-21
10 (Osali and Rostami, 2023)	Four-arm Parallel	Int:10 Ctrl:10	Metabolic syndrome	62.3 ± 1.23 Female	Int:33.56 ± 07.63 Ctrl:33.63 ± 04.54	Iran	6 weeks	80 mg/day nanocurcumin		Ctrl	BDI
11 (Saberi-Karimian et al., 2018)	Three arms DB, parallel	Int:37 Int:36 Ctrl:36	Metabolic syndrome	Int:40.05 ± 10.48 Int:37.52 ± 9.47 Ctrl:38.59 ± 10.28 Both	Int:30.66 ± 5.06 Int:30.67 ± 3.57 Ctrl:31.22 ± 4.67	Iran	6 weeks	1g/day cphospholipidated curcumin or curcumin		PBO	BDI, BAI
12 (Setiawati et al., 2017)	DB, parallel	Int:10 Ctrl:4	Systemic Lupus erythematosus	Int:29.7 ± 7.2 Ctrl:42.25 ± 15.65 Both	Int: 22.86 ± 3.38 Ctrl: 25.80 ± 2.40	Indonesia	4 weeks	50 mg/day curcuminoids		PBO	BDI
13 (Shafabakhsh et al., 2020)	DB, Parallel	Int:25 Ctrl:24	Type 2 diabetes and coronary heart disease	Int:64.9 ± 7.8 Ctrl:66.5 ± 7.7 Both	Int:30.3 ± 5.9 Ctrl:29.8 ± 2.9	Iran	12 weeks	1,000 mg/day curcumin		PBO	BDI
14 (Soltani et al., 2024)	DB, Parallel	Int:21 Ctrl:21	Coronary slow flow phenomenon	Int:54 ± 9 Ctrl:55 ± 8 Both	Int:29.7 ± 3.1 Ctrl:30.6 ± 3.8	Iran	12 weeks	80 mg/day nano-curcumin		PBO	BDI-II
15 (Yaikwawong et al., 2024)	DB Parallel	Int:113 Ctrl:114	Obese with Type 2 diabetes	Int:62.26 ± 8.82 Ctrl:62.26 ± 8.65 both	Int:26.76 ± 3.93 Ctrl: 27.21 ± 4.06	Thailand	12 months	1,500 mg curcuminoids		PBO	PHQ-9

Ctrl, control group; Tx, treatment group; DBP, double-blind parallel; Pts, participants; DB, Double-blind; PBO, placebo; Int, intervention; AU-NZ, australia and new zealand; NR, not reported. All included studies were of RCT, design.

#### 3.2.3 Risk of bias assessment

For depressive symptoms, two trials (Esmaily et al., 2015; Osali and Rostami, 2023) were judged to be at high risk of bias, seven trials (Kuszewski et al., 2020a; Lopresti et al., 2021b; Saberi-Karimian et al., 2018; Setiawati et al., 2017; Shafabakhsh et al., 2020; Hosseini et al., 2024) were considered to be at unclear risk of bias and seven trials (Yaikwawong et al., 2024; Soltani et al., 2024; Asadi et al., 2020; Hosseininasab et al., 2021; Lopresti et al., 2021a; Miodownik et al., 2019; Mojtahedi et al., 2024) at low risk of bias. No significant publication bias was found in the funnel plots or the Egger test (p = 0.888). For anxiety symptoms, four trials (Lopresti et al., 2021b; Saberi-Karimian et al., 2018; Shafabakhsh et al., 2020; Hosseini et al., 2024) were judged to be at unclear risk of bias and three trails (Asadi et al., 2020; Lopresti et al., 2021a; Mojtahedi et al., 2024) at low risk of bias. No significant publication bias was found in the funnel plots or the Egger test (p = 0.506).

#### 3.2.4 Depressive or anxiety symptoms

**FIGURE 3:**
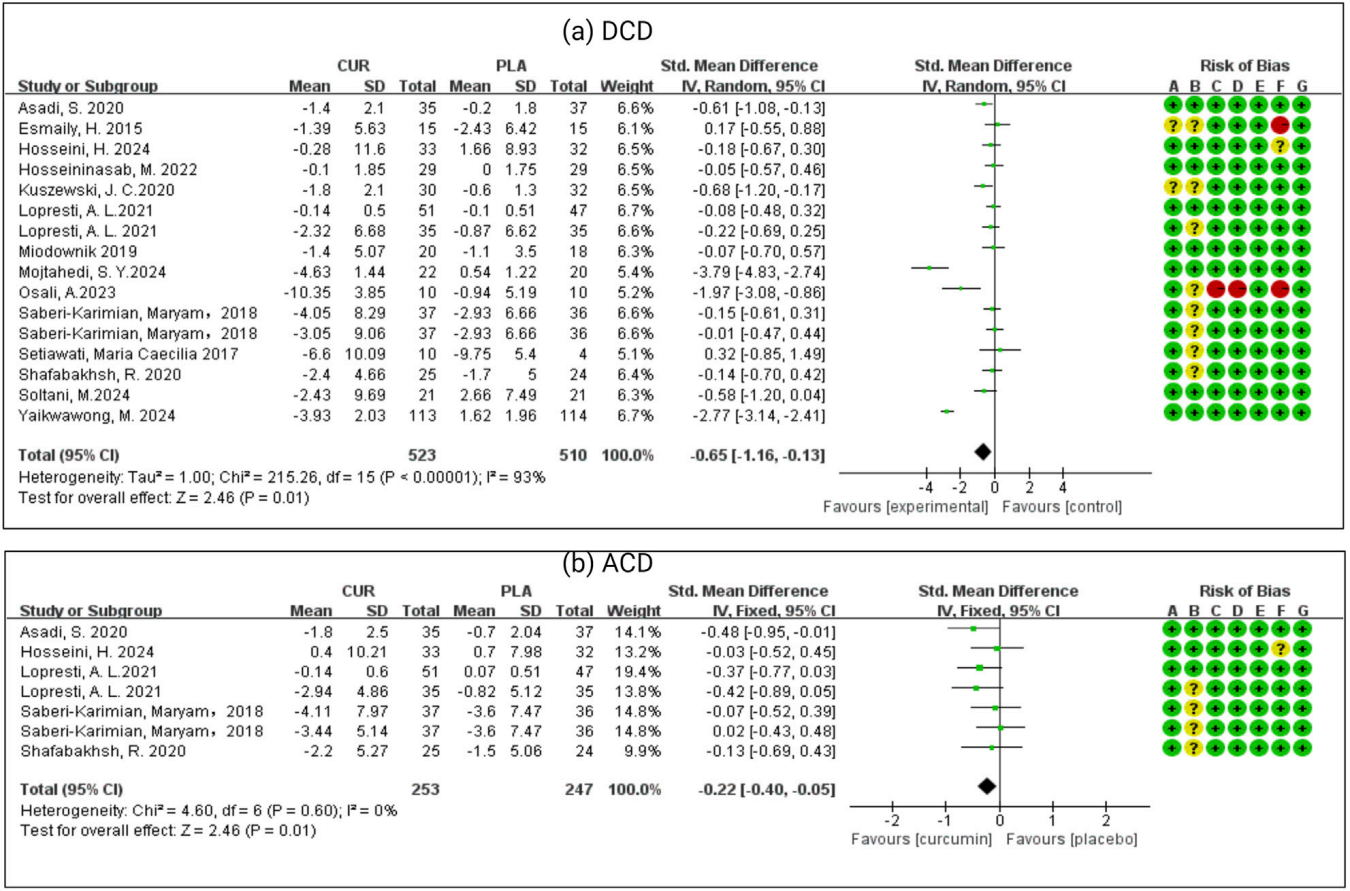
**(a)** Meta-analysis of patients who had depressive symptoms with chronic disease (DCD). **(b)** Meta-analysis of patients who had anxiety symptoms with chronic disease (ACD).

As we can see from Figure 3. Data relating to the depressive score’s outcome was available from all the RCTs. Curcumin group was better than the placebo group, with an SMD of −0.65 (−1.16, −0.13) in fifteen studies and 1,014 patients. There was significant heterogeneity in effect size (I^2^ = 93%, P < 0.0001). Data relating to the primary anxiety scores outcome was available from seven RCTs. Curcumin was better than the placebo, with an SMD of −0.22 (−0.40, −0.05) in seven studies and 494 patients. There was significant heterogeneity in effect size (I^2^ = 0%, P = 0.01).

**FIGURE 4:**
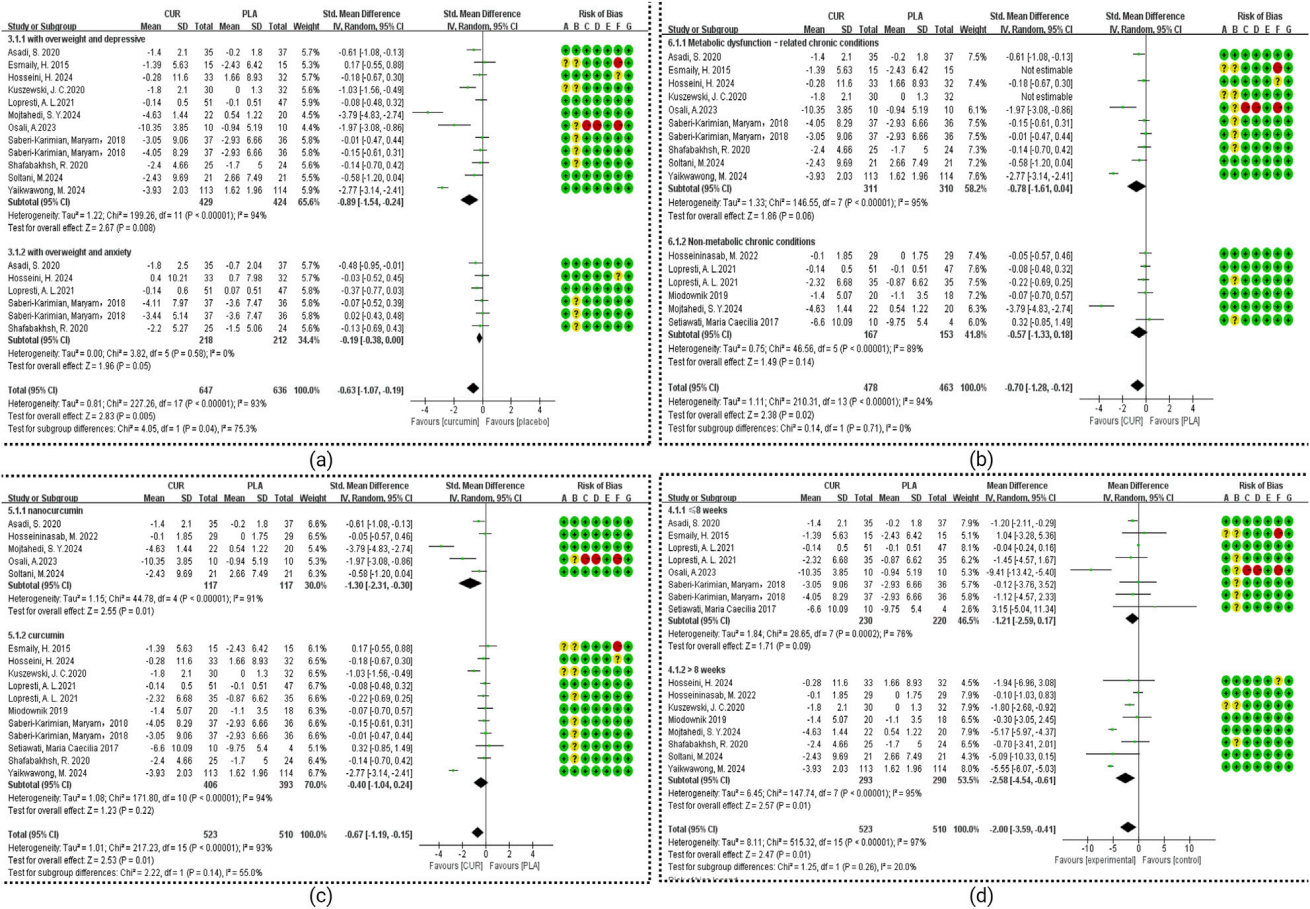
Meta-analysis of Subgroup. **(a)** Subgroup of BMI. **(b)** Subgroup of health status. **(c)** Subgroup of dosage form. **(d)** Subgroup of duration.

The subgroup analysis results as shown in Figure 4 and Table 2. Our analysis included seven studies examining anxiety symptoms with chronic disease (ACD) and six studies focusing on overweight populations (BMI subgroup). Statistical analysis for ACD in other subgroups was precluded due to insufficient studies (n < 3 per subgroup). Significant treatment effects were observed for the overweight with DCD group (SMD = −0.89, 95% CI: −1.54 to −0.24; I^2^ = 94%, p < 0.00001), and the overweight with ACD group (SMD = −0.19, 95% CI: −0.38 to 0.00; I^2^ = 0%, p = 0.58). Duration analysis revealed significant effects for interventions >8 weeks (SMD = −2.58, 95% CI: −4.54 to −0.61; I^2^ = 95%, p = 0.16) but not for shorter durations. Formulation-specific analysis demonstrated significant benefits for nanocurcumin (SMD = −1.30, 95% CI: −2.31 to −0.30; I^2^ = 91%, p = 0.01) but not for standard curcumin (SMD = −0.40, 95% CI: −1.04 to 0.24; I^2^ = 95%, p = 0.22). Health status stratification showed significant effects in metabolic conditions (obesity, diabetes, metabolic syndrome, coronary slow flow; SMD = −0.72, 95% CI: −1.41 to −0.03; I^2^ = 95%, p = 0.04) but not in other chronic diseases (SMD = −0.57, 95% CI: −1.33 to 0.18; I^2^ = 93%, p = 0.14).

**TABLE 2:** Pooled estimates of curcumin supplementation.

Group	SMD (95%CI)	P-value	*I* ^*2*^	P-heterogeneity
DCD	−0.65 (−1.16, −0.13)	0.01	93%	<0.0001
ACD	−0.22 (−0.40, −0.05)	0.01	0%	0.6
Overweight with DCD	−0.89 (−1.54, −0.24)	0.008	94%	<0.0001
Overweight with ACD	−0.19 (−0.38, −0.00)	0.05	0%	0.58
≤8 weeks	−1.21 (−2.59, 0.17)	0.09	76%	0.0002
>8 weeks	−2.58 (−4.54, −0.61)	0.01	95%	<0.0001
Nanocurcumin	−1.30 (−2.31, −0.30)	0.01	91%	<0.0001
Curcumin	−0.40 (−1.04, 0.24)	0.22	95%	<0.0001
Metabolic syndrome	−0.72 (−1.41, −0.03)	0.04	94%	<0.0001
others	−0.57 (-1.33, 0.18)	0.14	93%	<0.0001

Overweight with DCD, patients who were DCD, and overweight (BMI≥25). Overweight with ACD, patients who were ACD, and overweight (BMI≥25). ND, patients who had DCD, but lacked weight data or BMI <25.

#### 3.2.5 Drop-out and adverse events

**FIGURE 5:**
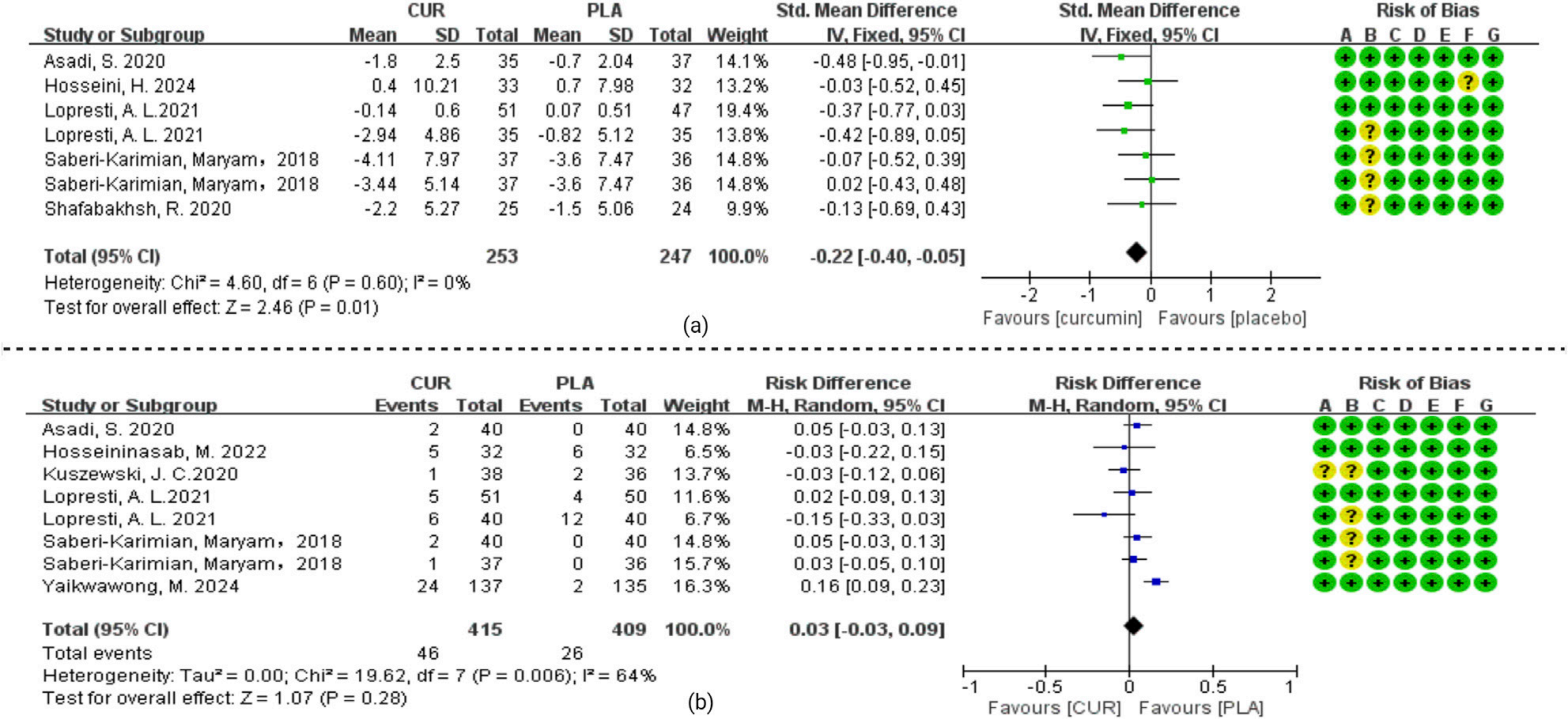
**(a)** Meta-analysis of curcumin *versus* placebo for patients with drop-out rates. **(b)** Meta-analysis of curcumin *versus* placebo for patients with adverse events.

Three studies (Esmaily et al., 2015; Osali and Rostami, 2023; Setiawati et al., 2017) were excluded from the forest plot analysis. This exclusion was based on the following rationale: two studies (Esmaily et al., 2015; Setiawati et al., 2017) reported zero dropouts, while the third study (Osali and Rostami, 2023) failed to provide any dropout data. As illustrated in Figure 5, our meta-analysis revealed no statistically significant difference in dropout rates between the curcumin and placebo groups (OR = 0.95, 95% CI 0.66 to 1.38, I^2^ = 0%, p = 0.80).

Safety data analysis revealed the following findings: Four articles (Esmaily et al., 2015; Osali and Rostami, 2023; Setiawati et al., 2017; Hosseini et al., 2024) failed to report any adverse event information. Another four trials (Soltani et al., 2024; Miodownik et al., 2019; Mojtahedi et al., 2024; Shafabakhsh et al., 2020) documented no adverse events in either treatment arm throughout the study duration. Among the remaining studies, gastrointestinal disturbances including abdominal pain, diarrhea, stomachache, and nausea were reported in seven trials (Yaikwawong et al., 2024; Asadi et al., 2020; Hosseininasab et al., 2021; Kuszewski et al., 2020a; Lopresti et al., 2021a; Lopresti et al., 2021b; Saberi-Karimian et al., 2018). Neurological symptoms such as headaches and daytime drowsiness were observed in five studies (Yaikwawong et al., 2024; Hosseininasab et al., 2021; Lopresti et al., 2021a; Lopresti et al., 2021b; Saberi-Karimian et al., 2018). As shown in Figure 5, meta-analysis demonstrated no statistically significant difference in adverse event incidence between curcumin and placebo groups (OR = 0.03, 95% CI: −0.03 to 0.09; I^2^ = 64%, p = 0.28). Importantly, all reported adverse effects were mild in nature, with no serious adverse reactions attributed to curcumin administration in any of the included trials.

#### 3.2.6 Sensitivity analysis

The leave-one-out analysis demonstrated notable variations in pooled estimates. For anxiety with chronic disease (ACD), the removal of one article (Mojtahedi et al., 2024) resulted in a substantial reduction in heterogeneity, with the I^2^ value decreasing from 84% to 0%, while the treatment effect remained statistically significant, changing from SMD -0.32 (95% CI: −0.50, −0.15) to SMD -0.22 (95% CI: −0.40, −0.05). Despite the study’s high quality and low risk of bias, its considerable influence on heterogeneity justified its exclusion from the final ACD analysis.

A comparable pattern emerged for depressive symptoms with chronic disease (DCD), where excluding one article (Yaikwawong et al., 2024) reduced heterogeneity from 93% to 78%. Although both values indicated high heterogeneity, the treatment effect persisted, shifting from SMD -0.65 (95% CI: −1.16, −0.13) to SMD -0.44 (95% CI: −0.75, −0.12). Besides, as shown in Figure 4, this article included different subgroup analyses (intervention duration, dosage form, health condition, and BMI), and the heterogeneity was above 90%. Since most studies did not include depression/anxiety patients of the same severity, it was difficult to conduct subgroup analysis based on baseline disease severity. And we analyzed the reason why this article had significant differences compared to other research findings, maybe cause the sample size (227 participants) of this article was approximately 3–5 times that of other studies and a long follow-up period (12 months), resulting in higher accuracy.

A comparable pattern emerged for depressive symptoms with chronic disease (DCD), where excluding one article (Yaikwawong et al., 2024) reduced heterogeneity from 93 percent to 78 percent. Although both values indicated high heterogeneity, the treatment effect persisted, shifting from SMD -0.65 (95% CI: −1.16, −0.13) to SMD -0.44 (95% CI: −0.75, −0.12). This study was retained in the DCD analysis due to its rigorous methodology, with its larger sample size (227 participants) and extended 12-month follow-up period likely contributing to the observed differences in effect size.

For adverse events was same, the same article (Yaikwawong et al., 2024) was not included in the statistics, then the result changed from OR = 0.03, CI 95%: −0.03, 0.09 I^2^ = 64% to OR = 0.01, 95% CI: −0.03, 0.06 I^2^ = 23%. However, both statistical results indicate no significant difference in adverse events between the curcumin group and the placebo group. This article has a large sample size (227 participants) and a long follow-up period (12 months), which may be the reason why the control group has more adverse events than other clinical trials.

## 4 Discussion

This systematic review and meta-analysis evaluated the efficacy and acceptability of curcumin in alleviating depressive and anxiety symptoms associated with chronic diseases. Emerging evidence from preclinical and clinical studies supports the therapeutic potential of curcumin across a spectrum of chronic conditions, including type 2 diabetes, obesity, migraine, arthritis, cardiovascular disease, and gastrointestinal disorders (such as IBS and IBD). By summarizing data from the animal literature, we conclude the mechanisms of curcumin for chronic diseases combined with anxiety/depressive are mainly focused on the following aspects: (1) Anti-inflammatory and antioxidant effects: for diabetes, obesity or pain, curcumin can reduce pro-inflammatory cytokines (TNF-α, IL-6) and regulate pathways such as NF-κB. For MI, curcumin can inhibit NLRP3 inflammasomes by reducing oxidative stress, mitochondrial dysfunction and reactive oxygen species (ROS), and inhibits CRP production in aldosterone-induced rat vascular smooth muscle cells through the ROS-ERK1/2 signaling pathway. The neuroprotective effects of curcumin are particularly evident in Alzheimer’s disease, where it addresses multiple pathological features including cholinergic system dysfunction, oxidative damage, and broader nervous system impairment. Similar benefits are observed in Toxoplasma gondii infection, where curcumin’s ability to reduce ROS levels helps alleviate depressive symptoms while its anti-inflammatory action provides additional therapeutic value.

(2) Energy metabolism: a. Insulin metabolism. Curcumin can reverse insulin resistance or protect RINm5F pancreatic β cells from apoptosis induced by nortriptyline by inhibiting the phosphoinositide 3-kinase/AKT/FOXO1 pathway and AKAP150/PKA/PP2B interaction, acting on diabetes caused by antidepressants. b. Lipid metabolism. In trigeminal neuralgia (TN), curcumin alleviates chronic pain-related depression by regulating lipid and glycerophospholipid metabolism. This metabolic modulation may contribute to its antidepressant effects in neuropathic pain conditions. (3) Regulating neurotransmitters to protect nerve cells. For post-stroke depression, curcumin activates the BDNF/Trkβ signaling pathway by enhancing the GAS5. Curcumin upregulates GAS5, which can reduce miR-10b and thereby reduce BDNF mRNA levels, or it may increase serotonin (5-HT) and dopamine levels and inhibit monoamine oxidase (MAO-A and MAO-B). For irritable bowel syndrome (IBS), curcumin normalizes neurotransmitter, BDNF and p-CREB levels in the hippocampus and colon. For seizures, curcumin may regulate central monoamine neurotransmitters and inhibit nitrosative stress and acetylcholinesterase activity. For neuropathic pain, curcumin may normalize depressive-like behavior through the serotonergic system in the spinal cord and downstream GABAA receptors. For Parkinson’s disease, the possible mechanism of curcumin is to enhance autophagy by promoting TFEB nuclear transport. (4) Modulation of the intestinal flora: For IBD, curcumin can increase phosphatidylcholine in the prefrontal cortex of mice and affect the MGB axis by regulating specific intestinal flora (mainly Musaceae may be intestinal flora), alleviating DSS-induced anxiety-like behavior. Overall, the literature suggests that curcumin exerts its beneficial effects through modulation of various signaling pathways, including NF-κB, NLRP3, AKAP150/PKA/PP2B, miR-146a-5p/ERK, BDNF/Trkβin, ROS-ERK1/2, GABAA receptor, gut-brain axis and Keap1-Nrf2-ARE, in different disease models and conditions. This convergence of evidence strengthens the rationale for curcumin’s use in managing depression and anxiety associated with chronic diseases. We propose that curcumin exerts its broad therapeutic effects via a unified “inflammation–oxidative stress axis”, primarily by inhibiting NF-κB and NLRP3 activation, reducing ROS and pro-inflammatory cytokines (TNF-α, IL-1β/18), as seen across models of depression, metabolic disorders, and neurodegeneration. Concurrently, curcumin enhances neuroplasticity by upregulating BDNF/TrkB signaling and activating ERK/CREB pathways, thereby reversing synaptic deficits and promoting neural repair. Moreover, curcumin modulates the gut–brain axis—it reshapes microbiota composition, strengthens intestinal and blood–brain barriers, and prevents bacterial endotoxins like LPS from triggering central inflammation. Through this tripartite mechanism—anti-inflammatory/antioxidant action, neuroplasticity support, and gut–brain communication—curcumin addresses the shared pathophysiology underlying chronic metabolic, neurological, and mood disorders. These converging pathways suggest that future interventions might achieve enhanced efficacy by simultaneously targeting inflammation, neural repair, and microbiota balance.

About half of the studies had a low risk of bias; the rest were high or unclear, which was important when interpreting results. Curcumin significantly reduced depressive symptoms in chronic disease (SMD = −0.65, 95% CI: −1.16 to −0.13) and anxiety (SMD = −0.22, 95% CI: −0.40 to −0.05). Effects were powerful for depression in individuals with BMI ≥25 kg/m^2^. Although anxiety outcomes were borderline (SMD = −0.19, CI: −0.38 to 0.00), they hint at a modest benefit. It is important to note that among the included studies, only two explicitly reported BMI <25 participants (i.e., non-overweight/not obese). In contrast, the other two studies did not provide BMI data. Consequently, we are unable to perform a subgroup analysis for the BMI <25 population with concurrent depressive or anxiety symptoms, due to the lack of BMI-specific information. These findings likely reflect curcumin’s anti-inflammatory and insulin-sensitizing actions in obesity-linked conditions. This aligns with epidemiological evidence showing that higher BMI predicts poorer quality of life and greater mood disturbance severity (Rindler et al., 2023). Mechanistic studies suggest these benefits may stem from curcumin’s dual ability to reduce inflammation and enhance insulin sensitivity in obesity-related conditions. Future research should aim to clearly report BMI distributions to facilitate more comprehensive evaluations of interventions in the normal-weight (BMI <25) population with psychological comorbidities.

Subgroup analyses found that curcumin’s antidepressant effects were duration-dependent: interventions over 8 weeks yielded significant benefits (SMD = −2.58, 95% CI: −4.54 to −0.61), whereas shorter courses (≤8 weeks) did not (SMD = −1.21, 95% CI: −2.59 to 0.17). This aligns with previous reports indicating time-dependent anxiolytic effects (Ruan et al., 2023). Moreover, formulation matters: nanocurcumin produced a substantial antidepressant effect (SMD = −1.30, 95% CI: −2.31 to −0.30), while conventional formulations had negligible impact (SMD = −0.40, 95% CI: −1.04 to 0.24), highlighting the importance of bioavailability-enhanced forms. Unfortunately, among the five studies included that evaluated nano-curcumin, only one provided categorical depression change data (e.g., proportions shifting between severity levels). The other four did not report transitions in depression severity, only pre- and post-intervention mean scores and SD values. Furthermore, depression was not the primary focus of these chronic disease studies, and depression scales (e.g., DASS-21, BDI-II) were not universally or uniformly applied across studies. Consequently, we cannot derive the proportion of participants improving by one severity category or estimate clinically meaningful change thresholds (e.g., moving from moderate to mild depression) for subgroup/meta-analysis. This limitation prevents reliable translation of our SMD findings into categorical clinical endpoints. Recent studies (Bertoncini-Silva et al., 2024; Ataei et al., 2023; Maleki Dizaj et al., 2022) noted that nano-formulations of curcumin substantially enhance its solubility, absorption, cellular uptake, and tissue distribution, leading to markedly increased bioavailability in various conditions, including neurodegenerative, cardiovascular, and metabolic disorders. The conclusions of these studies were also consistent with the statistical results of our article. Curcumin’s efficacy in treating depression appears to be closely associated with both the duration of treatment and the formulation used. Therefore, future research should focus on identifying the optimal treatment protocols (including duration) and formulation type (to maximize curcumin’s therapeutic potential) in depression management.

Recent mechanistic and epidemiological evidence indicates that conditions such as obesity, type 2 diabetes mellitus, hypertriglyceridemia, coronary heart disease, coronary slow-flow phenomenon, and diabetic peripheral neuropathy share common pathophysiological pathways, most notably insulin resistance, chronic inflammation, lipid metabolism disturbances, and endothelial dysfunction. Based on these overlapping mechanisms, we grouped these conditions into a single “Metabolic dysfunction–related chronic conditions” (MetSR) category. In contrast, studies involving conditions such as schizophrenia, osteoarthritis, migraine, and functional gastrointestinal disorders were grouped under “Non-metabolic chronic conditions”, due to their distinct primary pathophysiological pathways, which do not centrally involve metabolic dysregulation. Patients with MetSR demonstrated statistically significant improvement in depressive symptoms (DCD: SMD = −0.72, 95% CI: −1.41 to −0.03), while non-MetSR conditions showed no significant treatment effect (SMD = −0.57, 95% CI: −1.33 to 0.18). The pro-depressant effects of MetSR may be mediated through chronic low-grade inflammation, as evidenced by recent research showing significant mediation (30.8% combined effect) through inflammatory markers including platelets, lymphocytes, neutrophils, C-reactive protein (CRP), leukocytes, and INFLA scores (Cen et al., 2024). Supporting this mechanism, Qiu et al. (2023) reported that curcumin significantly improved several Metabolic Syndrome-associated inflammatory markers, including tumor necrosis factor-α (TNF-α), CRP, and malondialdehyde (MDA), though interleukin-6 (IL-6) and high-sensitivity CRP (hsCRP) showed no significant changes. These anti-inflammatory effects of curcumin in Metabolic Syndrome patients have been consistently observed in clinical studies (Gorabi et al., 2022).

The meta-analysis found no significant difference in dropout rates between curcumin and placebo groups (OR = 0.95, 95% CI: 0.66–1.38). Analysis of adverse effects (OR = 0.03, 95% CI: −0.03–0.09) revealed comparable safety profiles, with reported events primarily consisting of gastrointestinal symptoms (abdominal pain, nausea, reflux, diarrhea, constipation, flatulence, and indigestion), neurological effects (headache and daytime drowsiness), and occasional hypersensitivity reactions or cold sores. Notably, curcumin has demonstrated generally good tolerability even at high doses up to 12 g/day in clinical studies. However, some adverse effects, including diarrhea, headache, and changes in liver enzymes, have been reported at higher doses (Hewlings and Kalman, 2017). Most adverse effects were mild to moderate in severity, with no treatment discontinuations directly attributed to curcumin use. Comparative clinical trials have shown similar safety profiles between curcumin and established supplements like fish oil (Dennehy, 2011; Kuszewski et al., 2020b). These collective findings support curcumin’s favorable safety and tolerability as an adjunct therapy for depressive and anxiety symptoms in patients with chronic diseases.

Our updated meta-analysis demonstrates that curcumin supplementation significantly improves depressive and anxiety symptoms compared to control groups. Subgroup analyses revealed several important patterns: (1) treatment duration (<8 weeks vs. ≥ 8 weeks) did not significantly influence outcomes, suggesting the antidepressant effects may not be time-dependent; (2) nano-formulated curcumin showed superior efficacy compared to standard formulations; and (3) enhanced therapeutic benefits were observed in patients with higher BMI or Metabolic dysfunction–related chronic conditions disease. These findings highlight the need for future research to specifically investigate high-bioavailability curcumin formulations, particularly for managing mood symptoms associated with chronic metabolic disorders. The promising results in overweight populations suggest curcumin may offer targeted benefits for inflammation-related mood disturbances commonly seen in metabolic conditions.

The potential benefits of curcumin for managing depressive and anxiety symptoms associated with chemotherapy side effects, heavy metal toxicity, post-stroke conditions, myocardial infarction, trigeminal neuralgia, Parkinson’s disease, Alzheimer’s disease, epilepsy, and Toxoplasma gondii infection remain unverified in clinical trials. This evidence gap may reflect either a lack of complete studies or unpublished clinical data. Like previous meta-analyses examining curcumin’s effects on mood disorders (Xie and Deng, 2017; Gordon et al., 2018), the current review encountered substantial heterogeneity among included studies. While the adverse events heterogeneity could be attributed to one particularly sensitive study, the remaining variability likely stems from methodological differences in study design, participant characteristics, and curcumin formulations (including bioavailability-enhancing technologies). These limitations necessitate cautious interpretation of our pooled results.

## 5 Limitation

A key limitation of this review lies in the considerable heterogeneity among the included studies, particularly in the analysis of depressive symptoms. For instance, excluding one study with a large sample size and long follow-up duration reduced heterogeneity from 93% to 78%, yet both levels remained high. This heterogeneity may stem from differences in intervention duration, baseline health conditions, as well as the varying severity of depression and anxiety across study populations, which limited our ability to perform subgroup analyses based on baseline disease severity. Additionally, several individual trials had small cohorts, which may reduce statistical power and affect the generalizability of the findings. Evidence from non-metabolic chronic diseases—such as inflammatory bowel disease, neurodegenerative disorders, and autoimmune conditions—remains sparse, restricting broader conclusions. Furthermore, approximately half of the included trials were assessed as having a high or unclear risk of bias, which may compromise the overall reliability of the pooled results.

## 6 Conclusion

Current evidence indicates that Curcuma longa and its bioactive compounds may offer potential benefits in alleviating symptoms of depression and anxiety in patients with chronic diseases, particularly those with metabolic dysfunction-associated conditions (DACD). However, due to high heterogeneity among studies and generally short intervention durations, these findings should be interpreted cautiously. Moreover, evidence in non-metabolic chronic conditions, such as inflammatory bowel disease, neurodegenerative disorders, and autoimmune diseases, remains limited. Therefore, further high-quality randomized controlled trials are needed to confirm these preliminary findings and to evaluate the efficacy of Curcuma longa in a broader range of chronic illnesses.

## Data Availability

The original contributions presented in the study are included in the article/supplementary material, further inquiries can be directed to the corresponding authors.
