# Supplementary figures and images for: Potential therapeutic benefits of curcumin in depression or anxiety induced by chronic diseases: a systematic review of mechanistic and clinical evidence
Source: Front Pharmacol. 2025 Aug 22;16:1638645. doi: 10.3389/fphar.2025.1638645 (PMC12411784; doi:10.3389/fphar.2025.1638645)

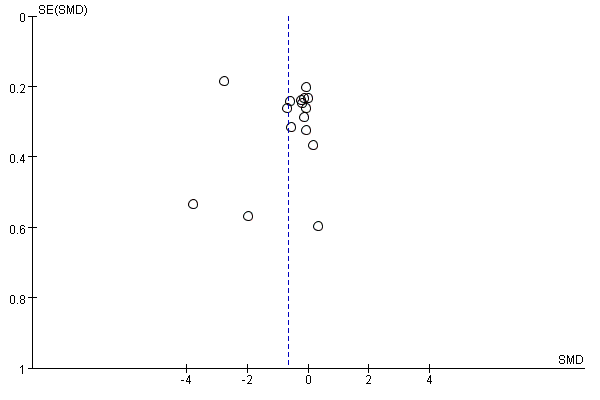


Funnel plot for DCD


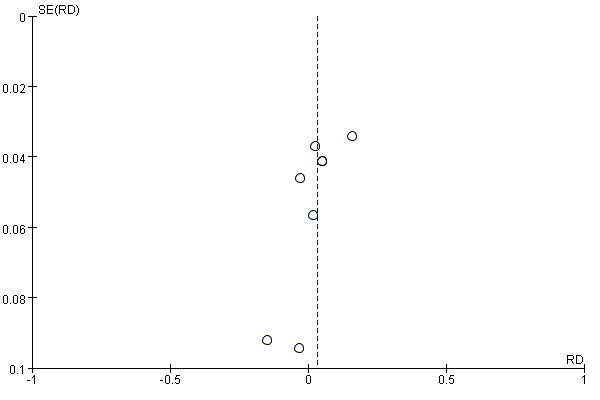


Funnel plot for adverse

Egger test for adverse

Supplement: Supplementary file 1 [file Supplementaryfile1.doc]
